# Macrophages and Dendritic Cells Are the Predominant Cells Infected in Measles in Humans

**DOI:** 10.1128/mSphere.00570-17

**Published:** 2018-05-09

**Authors:** Ingrid V. Allen, Stephen McQuaid, Rosana Penalva, Martin Ludlow, W. Paul Duprex, Bertus K. Rima

**Affiliations:** a Centre for Experimental Medicine, School of Medicine, Dentistry and Biomedical Sciences, Queen’s University Belfast, Belfast, Northern Ireland; b Tissue Pathology Laboratories, Belfast Health and Social Care Trust, Belfast, Northern Ireland; Icahn School of Medicine at Mount Sinai

**Keywords:** B cells, T cells, dendritic cells, human infection, macrophages, measles

## Abstract

Characterization of human measles cases is essential in order to better assess the data generated in model systems of morbillivirus infection. To this end, we collected formalin-fixed tissue samples from 23 natural measles cases from different areas in the world and different phases of disease ranging from prodromal and acute measles to a persistent infection in an immunocompromised subject. We show that the vast majority of measles virus (MV)-infected cells in epithelia were intraepithelial immune cells that were, in most cases, positive for the CD11c myeloid cell marker. Small numbers of measles virus-infected cytokeratin-positive epithelial cells were also detected in bronchial and appendix epithelia. Dissolution and disruption of uninfected and MV-infected alveolar and bronchial epithelia were prominent features of the measles cases, especially in the established and late phases of the disease. In some instances, this was associated with the formation of MV-infected multinucleated giant cells which expressed CD11c and/or macrophage cell marker 68, a pathological feature also prominently observed in closely associated mucosa-associated lymphoid tissue. Collectively, these data show that resident and inflammatory infiltrating immune cells alter the architecture of respiratory tract epithelia and highlight the necessity for additional research into the function(s) and expression of nectin-4 in human tissues.

**IMPORTANCE** We have brought together a unique collection of 23 human cases of measles infection and studied the types of cells that are infected. This work has not been done with modern technologies such as double labeling with antibodies and confocal microscopy in human cases primarily due to the fact that it is difficult to obtain the material because, fortunately, measles is fatal in only a very small fraction of infected patients. During the past decades, the receptors for measles virus have been elucidated and monkey models have been developed. We found that, in most cases, independently of whether the tissues were obtained early or later in the infection, the primary cell types that were infected were those of the immune system such as lymphocytes, macrophages, and dendritic cells. A very small number of epithelial cells were also found to be infected.

## Abstract (precis)

We have brought together a unique collection of 23 human cases of measles infection and studied the types of cells that are infected. This work has not been done with modern technologies such as double labeling with antibodies and confocal microscopy in human cases primarily due to the fact that it is difficult to obtain the material because, fortunately, measles is fatal in only a very small fraction of infected patients. During the past decades, the receptors for measles virus have been elucidated and monkey models have been developed. We found that, in most cases, independently of whether the tissues were obtained early or later in the infection, the primary cell types that were infected were those of the immune system such as lymphocytes, macrophages, and dendritic cells. A very small number of epithelial cells were also found to be infected.

## INTRODUCTION

Despite the historical importance of measles virus (MV) as a major cause of childhood morbidity and mortality, the mechanisms by which the virus causes disease in humans remain poorly defined. Significant progress has been made in recent years toward reducing measles mortality levels and, ultimately, toward MV elimination, but it remains a major cause of death worldwide, having been responsible for approximately 145,700 deaths in 2013 (1). MV continues to cause significant outbreaks, even in the developed world, particularly where vaccination coverage falls below 90% of the population (2, 3). MV, with an estimated reproduction rate (R0) of 12 to 18, is the most infectious human virus and is primarily transmitted by aerosols from an infected host to the upper respiratory tract of susceptible individuals (2, 4). The first and least clinically relevant receptor identified for MV was the membrane cofactor protein (MCP; CD46) (5, 6). However, this is used *in vitro* only by laboratory-adapted and vaccine strains of MV (7). The primary cellular receptor for MV and related animal morbilliviruses is signaling lymphocytic activation molecule family member 1 (SLAM/F1; CD150) (8), which is used by wild-type, vaccine, and laboratory-adapted strains. Expression of CD150 is restricted to cells of the immune system, hematopoietic stem and progenitor cells (9), and platelets (10, 11). Subsequently, evidence suggested that an additional MV receptor(s) is present on epithelial, endothelial, and neuronal cells (12–14). This resulted in the discovery that nectin-4 (PVRL4) is a cellular receptor for MV at the adherent junctions of epithelial cells (15, 16). Assessment of the *in vivo* distribution of these receptors thus raised the issue of which cell types are infected during disease progression, i.e., of whether they are cells of the immune system or epithelial cells or both. *In vitro* and *in vivo* experimental evidence established CD150 as the primary cellular receptor, expression of which is critical for productive infection with wild-type MV strains and for cell-to-cell spread in the host (17, 18). Those conclusions are supported by both *in vitro* work in human dendritic cells (DCs) and peripheral blood lymphocytes (19) and *in vivo* studies in macaques (20, 21). Our previous nonhuman primate studies have provided a number of insights into measles pathogenesis and were designed to emulate natural measles virus infection, since virus was administered by the respiratory route. In the early stages of infection in the macaque, MV predominantly infects DCs and alveolar macrophages in the deep lung (22) prior to trafficking to regional lymph nodes (LNs), where the infection is amplified in CD150^+^ lymphocytes. Support for the idea of a role for DCs in measles pathogenesis was provided by an earlier *in vitro* study by de Witte and coworkers in which the authors demonstrated that CD150 and C-type lectin DC-SIGN, which facilities virus transfer to T-lymphocytes, are both involved in DC infection and subsequent spread of *de novo* synthesized virus (19).

The issue of which cells are infected by MV during the course of the human infection led us to perform a comprehensive study into the pathology of measles using a unique collection of human tissues representing different phases of the disease. Although many *in vitro* and *in vivo* models have been published previously, comprehensive analyses of human cases to establish the phenotype of MV-infected cells and their receptor status are lacking. We recognize the inherent challenges in such a study since we are limited to a subset of rare human cases in which measles was largely the cause of death. Nevertheless, the strength of this study is the direct applicability to natural measles in humans and the fact that it encompassed a unique collection of 21 autopsy and 2 biopsy cases of measles that spanned 38 years in clinical presentation and were of a wide range of geographical origins. Only cases in which the presence of an MV protein(s) could be demonstrated in one or more organs were included. The phenotype and MV receptor status of infected cells were determined in a wide variety of tissues, including respiratory epithelium tissue due to its importance in viral transmission. We report the identification of large numbers of MV-infected cells in epithelia, the majority of which were of a myeloid lineage and were associated with the formation of multinucleated giant cells (MGCs) and disruption of epithelia.

## RESULTS

### MV produces a consistent pathological response, irrespective of coexisting disease and date and place of presentation.

**FIG 1:**
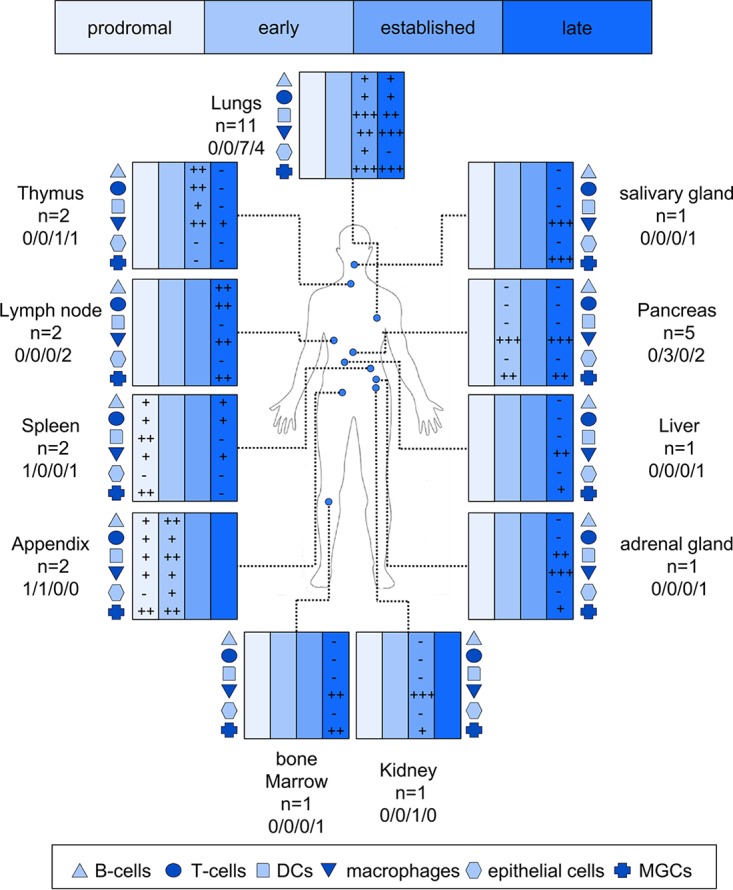
A schematic diagram showing the cellular tropism of MV and the degree of cell-to-cell fusion in organs from measles patients. MV antigen levels in B and T cells, dendritic cells (DCs), macrophages, epithelial cells, and multinucleated giant cells (MGCs) were assessed based on dual-labeling immunostaining and morphology of infected cells and were classified according to a semiquantitative scoring system as follows: −, no infected cells; +, 1 to 20 infected cells per section; ++, <20 foci of infection per section; +++, >20 foci of infection per section. The number of samples analyzed per organ is indicated beneath the name of the organ, and the data are further segregated into how many samples were analyzed for each of the four stages of measles (prodromal, early, established, and late).

Samples from twenty-three cases were obtained from Africa, the Americas, Asia, and Europe. The cellular pathological response specific to measles was assessed after H&E staining. Tissue blocks showing abnormal histology as well as selected blocks showing normal histology were then stained for MV antigen. A range of tissues were chosen against data on patient age, sex, country, year of presentation, interval of rash onset to biopsy or death, and, where relevant, coexisting disease and cause of death (Table 1). Among 15 patients who had coexisting disease, 1 had an inherited immune disorder, 6 were human immunodeficiency virus type 1 positive (HIV-1^+^), 2 had been treated for lymphoma and 4 for leukemia, and 2 had congenital heart disease. In all of these cases, the pathological response was consistent in that macrophages and DCs were the predominant infected cells. Data for infected and noninfected tissues from a range of organs, set against the phase of the disease, were collated (Table 2) with a comprehensive summary of the virus cellular tropism of MV in these tissues (Fig. 1). The figure shows that B- and T-lymphocytes, macrophages, DCs, and, to a lesser extent, epithelial cells were all targets of MV infection.

**TABLE 1:** Clinical features and disease outcomes in 23 cases of measles

Phase of measles^a^	Case no.	Age (yrs)	Sex^b^	Country of presentation	Yr of presentation	Time from onset of rash to biopsy or to death	Coexisting disease	Disease outcome /cause of death
								
Prodromal	1	4	M	Northern Ireland	1970	No rash	Congenital heart disease	Congenital heart disease
	2^c^	12	M	South Korea	2000	No rash	None	Alive/well
								
Early	3^c^	10	F	South Korea	2000	<3 days	None	Alive/well
	4	3	M	Northern Ireland	1962	3 days	None	Staphylococcal bronchopneumonia
	5^c^	14	M	Brazil	1997	3 days	Treated lymphoma	Disseminated measles
								
Established	6	<1	F	Northern Ireland	1966	10 days	Congenital heart disease	Measles pneumonia/congenital heart disease
	7^c^	3.5	F	Côte d’Ivoire	1991	<15 days	HIV-1 positive	Measles tracheitis/MV^+^ mucus glands
	8^c^	2.5	F	Côte d’Ivoire	1991	<15 days	HIV-1 negative	MV pneumonia/MV^+^ in thymus
	9^c^	1.5	M	Côte d’Ivoire	1991	<15 days	HIV-1 negative	MV pneumonia/MV^−^ in thymus
	10^c^	1.0	M	Côte d’Ivoire	1991	<15 days	HIV-1 negative	MV pneumonia
	11^c^	<1	F	Côte d’Ivoire	1991	<15 days	HIV-1 negative	MV pneumonia
	12^c^	<1	M	Côte d’Ivoire	1991	<15 days	HIV-1 positive	MV pneumonia
	13^c^	1	F	Côte d’Ivoire	1991	<15 days	HIV-1 positive	MV pneumonia
	14^c^	0.6	F	Côte d’Ivoire	1991	<15 days	HIV-1 positive	MV pneumonia
	15^c^	0.6	F	Côte d’Ivoire	1991	<15 days	HIV-1 positive	MV pneumonia
	16^c^	1.2	M	Côte d’Ivoire	1991	<15 days	HIV-1 positive	MV pneumonia
								
Late	17^c^	4	M	Brazil	1997	20 days	Treated lymphoma	Disseminated measles
	18^c^	<1	M	Northern Ireland	1968	42 days	Genetic immune disease	Disseminated measles (vaccine induced)
	19^c^	5	M	United States	1965	42 days	Treated leukemia	Disseminated measles
	20^c^	5	M	United States	1971	180 days	Treated leukemia	Disseminated measles
	21^c^	6	M	United States	1971	180 days	Treated leukemia	Disseminated measles
	22^c^	68	F	Switzerland	1971	No rash	Treated leukemia	Disseminated measles
	23^c^	7	M	Northern Ireland	1989	49 days	Treated leukemia	Disseminated measles

a Definitions of phase of measles are based on the time interval between the time of the appearance of a rash and the time of biopsy or death. Prodromal, no rash; early, 1 to 3 days; established, 4 to 15 days; late, >15 days.

b F, female; M, male.

c Case published (23, 37, 61–63).

**TABLE 2:** Numbers of cases in which MV was demonstrated in specific tissues for each temporal phase of measles

Organ	No. of cases in which MV positivity was observed/no. of cases for which the specific tissue was available and studied			
	Prodromal (*n* = 2)^a^	Early (*n* = 3)^a^	Established (*n* = 11)^a^	Late (*n* = 7)^a^
Bronchi/lungs	0/1	0/2	11/11	6/6
Trachea	0/1	NA^b^	2/10	3/5
Appendix	1/1	1/1	NA	0/1
Spleen	1/1	0/2	0/11	1/4
Pancreas	0/1	1/2	0/10	2/5
Liver	0/1	0/2	0/11	1/3
Adrenal	0/1	0/2	0/10	1/2
Kidney	0/1	0/2	1/11	0/2
Thymus	0/1	1/1	3/11	3/3
Lymph nodes	0/1	NA	0/8	2/3
Brain	NA	NA	NA	1/1

a Numbers of cases available for study in each phase.

b NA, tissue not available.

### MV predominantly infects cells of the immune system, irrespective of the duration of infection. (i) Prodromal (cases 1 and 2).

**FIG 2:**
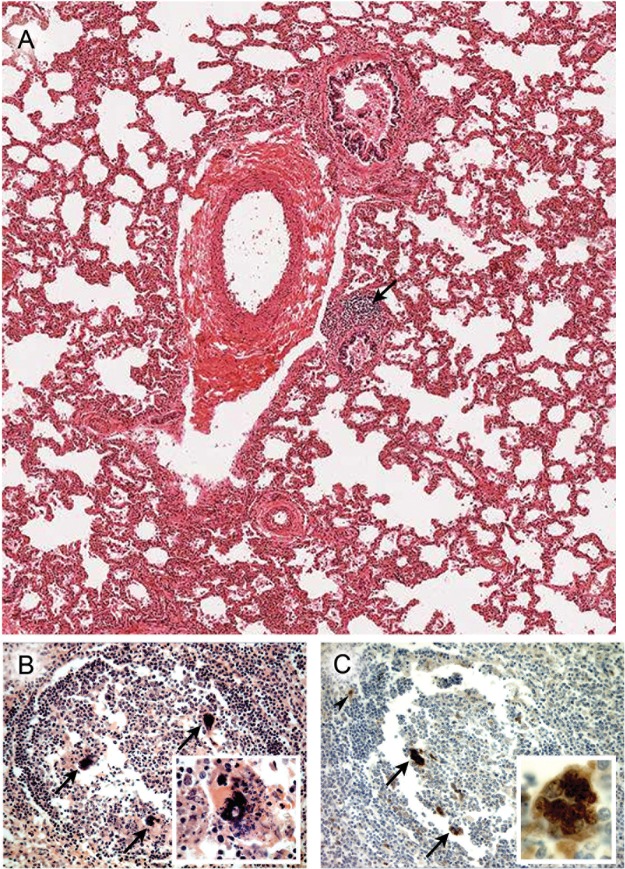
Lung and spleen in prodromal measles. (A) H&E-stained section of prodromal lung (case 1) showing normal architecture without alveolar inflammation. A lymphoid aggregate is seen around a bronchiole (arrow). (B) H&E-stained section of a lymphoid follicle in the white pulp of the spleen (case 1). Arrows indicate MGCs. Inset, H&E of MGC from an adjacent lymphoid follicle undergoing apoptosis or necrosis. (C) Immunohistochemical analysis of MV antigen in a serial section (case 1) of a lymphoid follicle shown in panel B. (The inset shows a section that was not a serial section.) The MGCs are indicated as follows: arrow, MV^+^; arrowhead, MV^+^ mononuclear cell. Original magnifications: panel A, ×25; panel B, ×250 (inset, ×400); panel C, ×250 (inset, ×1,000).

**FIG 3:**
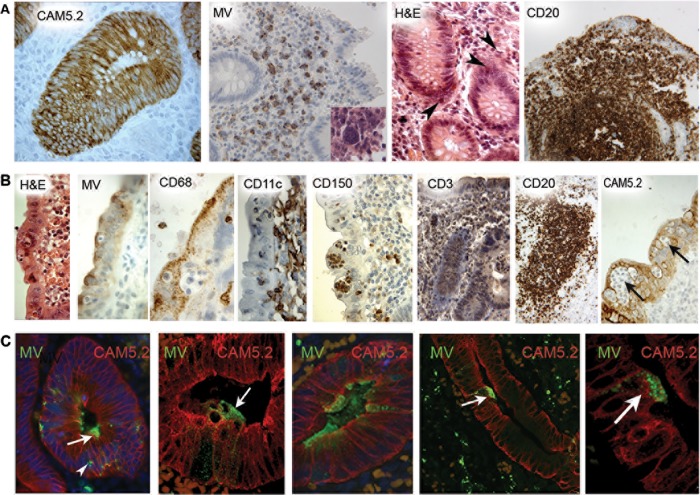
Appendix in prodromal and early measles. (A) Appendix in a prodromal case (case 2). The first (from the left) image shows an intact cerium ammonium molybdate (CAM)-stained crypt epithelium. The inset in the second (MV) image is an H&E-stained image with an MGC. The third (H&E-stained) image shows MGCs (arrows) in the lamina propria. The fourth image shows CD20^+^ B cells in the lamina propria, MALT, and epithelium. (B) Appendix of early case 3. The first images shows a surface epithelium with macrophages within the epithelium and at the luminal epithelial surface (H&E staining). The second image shows a punctate staining pattern of MV antigen in cells at the epithelial luminal surface. The third image shows CD68^+^ staining at the luminal surface of the epithelium, CD68^+^ cells in the lamina propria, and one unidentified CD68-negative cell in the epithelium. CD11c^+^ cells are mainly confined to the lamina propria. CD150^+^ cells are observed within the epithelium and lamina propria, whereas CD3-positive and CD20-positive cells are primarily located in the lamina propria. (C) Appendix of early case 3. All images show MV (green), CAM 5.2 (red), and nuclei (DAPI, blue). MV positivity is seen at the luminal epithelial surface and above the epithelial basement membrane (arrow). In the first image (left), showing an intraepithelial MV^+^ cell, a CAM 5.2^+^ focus is seen with many nuclei, possibly representing an infected MGC (arrow). MV positivity is seen at the luminal epithelial surface in the leftmost three images (arrow). In image 4 and, at higher magnification, image 5, MV infection is shown in a CAM 5.2^+^ cell traversing the epithelium. Original magnifications: panel A, ×25 (H&E), ×100 (H&E) (MV) (inset, ×250), ×250 (H&E), and ×100 (CD20); panel B, ×400 (MV, CD68; H&E), ×500 (CD150, CD11c), ×250 CD3, and ×100 CD20. In panel C, all images are ×400, except the first and fourth images, which are ×200.

Pathological assessments were performed on a range of tissues from the four phases of measles (Tables 1 and 2). In the first case (case 1) in the prodromal phase, which came to necropsy because of cardiac surgery, MV antigen was undetectable in tracheal, bronchial, or alveolar epithelium and the architecture of respiratory epithelia was normal (Fig. 2A). It has been long recognized that multinucleated giant cells (MGCs) are a hallmark of MV infection *in vitro* and *in vivo* and are caused by cell-to-cell fusion of adjacent cells. Occasional MGCs were observed within the bronchiolar lumen, but since these were absent in the parallel section stained for MV, whether the MGCs represented induction by virus remains unclear (data not shown). MV infection was confirmed in lymph nodes and thymus and in the white pulp of the spleen. In the spleen, MGCs were located toward the periphery of lymphoid follicles (Fig. 2B). MGCs and a few mononuclear cells were MV positive (Fig. 2C). The second prodromal case (case 2) involved a boy who made a full recovery after an appendectomy. There was no evidence of infection or of the presence of MGCs or measles-induced lesions in the epithelium. MV antigen was detected in mononuclear cells and MGCs in mucosa-associated lymphoid tissue (MALT) and in the lamina propria in the appendix (Fig. 3A). B cells were identified using antibodies to CD20 (a nonglycosylated phosphoprotein present on all mature B cells) and were present in the epithelium and in greater numbers in the lamina propria and in MALT (Fig. 3A).

### (ii) Early cases (cases 3 to 5).

Case 3 represented a patient who presented with acute appendicitis and who made a full recovery following treatment. A hematoxylin and eosin (H&E)-stained section of the appendix shows numerous inflammatory cells in the epithelium (Fig. 3B, left panel). Results of MV antigen and CD68 (a lysosomal membrane glycoprotein present in macrophages and some DCs) staining at the luminal surface (Fig. 3B, panels 2 and 3) were similar, suggesting that macrophages accumulate in appendicular epithelia in close association with active sites of MV infection. In order to extend the analysis, sections were stained with antibodies to CD150, CD3 (a marker of mature lymphocytes coupled to their T cell receptors), CD20, and CD11c (a type 1 transmembrane protein found at high levels in most human DCs but also expressed on macrophages, monocytes, neutrophils, and some B cells). Cytokeratin was detected in tissue sections with antisera to CAM 5.2 (an epitope found in both CK8 and CK18 epithelial cells and which in normal tissues reacts with secretory epithelium). CD150 staining demonstrated focal immune cells in the epithelium (Fig. 3B, panel 5). The CD11c, CD3, and CD20 staining showed evidence of positive cells in the epithelium, but these were more numerous in the underlying lamina propria and MALT (Fig. 3B, panels 4, 6, and 7 from the left). Staining for CAM 5.2 showed evidence of severe disruption of the normal architecture of the epithelium due to infiltration of cytokeratin-negative immune cells (Fig. 3B, panel 8, arrows).

Although single-marker immunohistochemical staining of cell-type-specific markers and MV-infected cells was helpful for screening the samples, it is not possible to determine from these data which cell type(s) was infected. Therefore, sections from the appendix (case 3) were doubly stained for MV and cellular markers. Viral antigen was detected in luminal and crypt epithelium predominantly at the luminal aspect (Fig. 3C, middle panel). MV antigen was observed to colocalize with low numbers of intact CAM 5.2-positive appendicular epithelial cells, but by far the greater amount of the MV antigen staining appeared be in cells in the lumen on top of the epithelium (Fig. 3C). MALT was heavily infected, and MV was observed to colocalize with CD11c and CD68 cells in these tissues (data not shown). Appendicular MALT was more heavily infected than the epithelium, and the presence of MV was demonstrated in MGCs and mononuclear cells in MALT and lamina propria (data not shown). In case 4, the patient developed staphylococcal bronchopneumonia and, despite antibiotics and hydrocortisone treatment, died 3 days after the initial onset of the measles symptoms. Measles virus antigen was present in the thymus but not in the spleen or in the alveolar and bronchial epithelium (data not shown). Histological examination of sections from both lungs for case 5 by the use of H&E staining showed striking inflammatory changes, with numerous macrophages and MGCs. MV antigen was present focally in the pancreas, mainly within ducts and in the periductal connective tissue, which also showed infiltration with CD68^+^ cells (see Fig. 6B).

### (iii) Established (cases 6 to 16).

**FIG 4:**
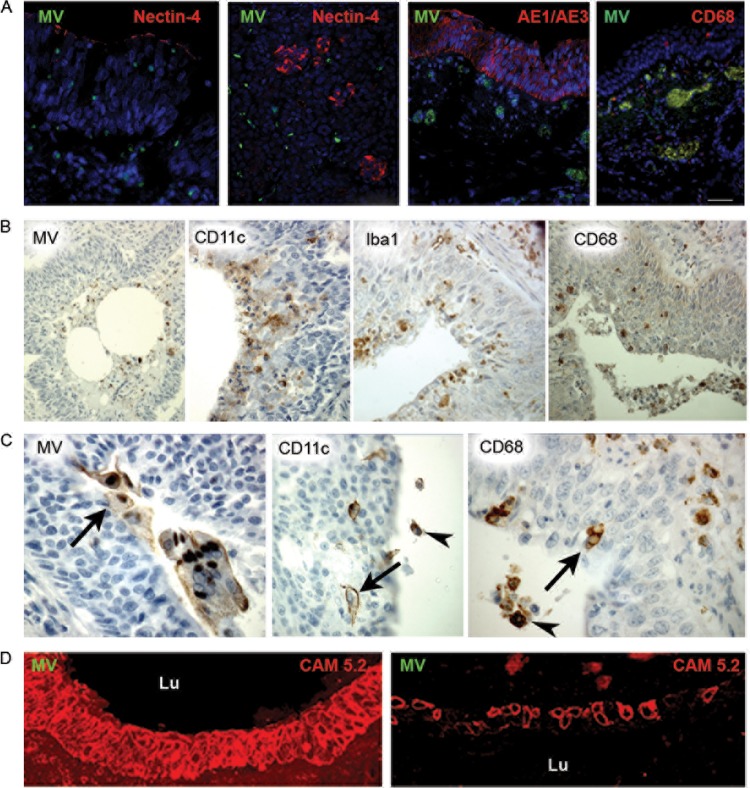
Trachea, bronchi, and bronchioles in established-measles pneumonia. (A) Sections visualized by CLSM were stained with antibodies against MV (green) and nectin-4, AE1/AE3, or CD68 (red). The images show MV, nectin-4, CD68, and AE1/AE3 staining in established case 9. As a positive control for MV and nectin-4 staining, pancreas tissue from established case 9 is shown in the second image from the left. (B) MV, CD11c, Iba1, and CD68 immunohistochemical analysis of the epithelial layer of bronchiole tissue from case 10. MV antigen-positive cells are observed partly within the bronchiolar lumen and partly at the luminal epithelial surface. CD11c^+^, Iba1^+^, and CD68^+^ cells are present at the luminal epithelial surface and within the epithelium. (C) Immunohistochemical analysis of MV, CD11c, and CD68 in the epithelial layer of a bronchus in established-measles pneumonia case 12. MV antigen-positive cells are seen on the epithelial surface (arrow). CD11c^+^ cells and CD68^+^ cells are seen in the lumen (arrow) and in the epithelium (arrow), sometimes outlining individual epithelial cells. (D) CLSM image of a section doubly stained for MV (green) and CAM 5.2 (red) from case 12 (same block as in panel C). Both images are from a bronchiolar epithelium close to an area of MV positivity. On the left, the epithelium is intact and is not infected. On the right, epithelial cells have lost their cohesion but are not infected. Lu, lumen. Original magnifications: panel A, ×400 (MV and nectin-4), ×400 (MV and AE1/AE3), and ×200 (MV and CD68); panel B, ×100 (MV) and ×250 (CD11c, Iba1, and CD68); panel C, ×625 (MV and CD68), ×500 (CD11c); panel D, ×500.

In all cases except two in which the infection was classified as established, there was a fatal outcome that was associated with measles pneumonia. Samples from the trachea showed occasional foci of inflammation with infiltration by lymphocytes and macrophages. Tracheal sections were examined using MV-, CD68-, AE1/AE3 (like the CAM 5.2a marker for epithelial cells)-, and nectin-4-specific antibodies (Fig. 4A). In all cases, MV antigen was undetectable in the tracheal epithelium, although a single focus of infection was observed on the epithelial surface in the sample from case 12. In one case, MV antigen was present in the subepithelial mucus glands. Sections from the pancreas were assessed (case 9). MV-infected cells did not colocalize with nectin-4^+^ or AE1/AE3^+^ cells; instead, MV^+^ and CD68^+^ cells were found in the connective tissue (Fig. 4A). The pancreas was positive for nectin-4 predominantly in the islets (Fig. 4A, panel 2). Sections from the thymus were assessed (cases 9 and 18). CD150^+^ cells colocalized with MV antigen (data not shown). In all of the MV-infected foci in bronchioles (Fig. 4B) and bronchi (Fig. 4C), CD11c^+^, CD68^+^, Iba1^+^ (ionized calcium-binding adapter molecule, expressed selectively in macrophages/microglia), and CD150^+^ cells were consistently found. These immune system cells often outlined epithelial cells (Fig. 4C). Bronchiolar subepithelial mucus glands frequently stained positively for MV and showed epithelial fragmentation and epithelial cell loss with replacement and luminal obliteration by CD11c^+^ cells (data not shown). Much of the bronchial and bronchiolar epithelium was not infected, and the cytokeratin was intact. However, a loss of intercellular cohesion was observed compared to uninfected epithelial cells within some bronchioles in MV-infected lungs (Fig. 4D). Necrotic epithelium and cytokeratin fragments, mixed with MV-infected cells, DCs, and macrophages, were present in bronchial and bronchiolar lumina, even though the lining epithelium appeared normal and was not infected (data not shown).

**FIG 5:**
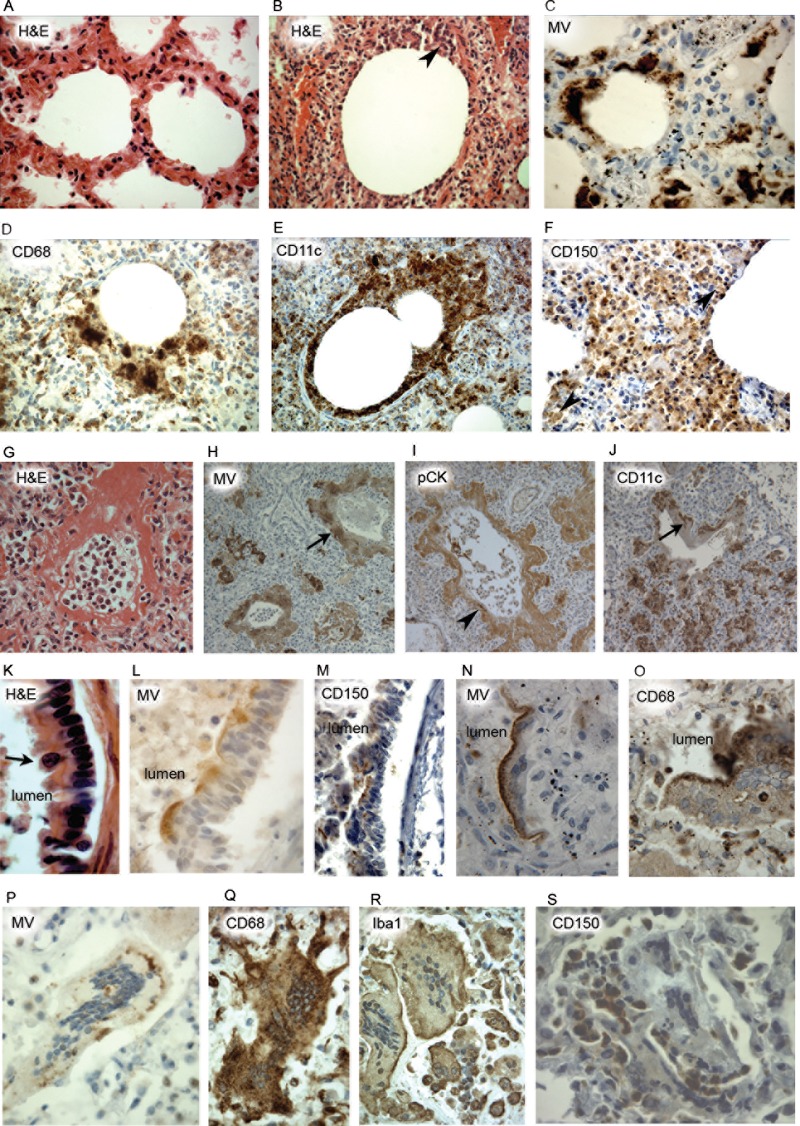
Immunohistochemical analysis of host response in alveoli and bronchioles in established-measles pneumonia. A noninflamed alveolus in tissue from prodromal-measles case 1 (A) is shown for comparison with an inflamed alveolus from established-measles pneumonia case 10 (B to F) in which the alveolar sac is partially obliterated by inflammatory cells (B) and the alveolar wall partly outlined by MV^+^ cells (C). (D) CD68^+^ cells partly outline an alveolus close to CD68 positivity in the interstitium. (E) CD11c^+^ cells outline the alveolus and are profuse in the interstitium. (F) CD150^+^ cells are seen in the interstitium and abut on the alveolar sac. (G) An H&E image of a hyaline membrane replacing the alveolar epithelium in established case 13. (H to J) Positivity within the hyaline membrane is seen for MV (arrow) (H), pan-cytokeratin (arrowhead) (I), and CD11c (arrow) (J) in case 13. (K) Sections of a bronchiole stained with H&E in established-measles pneumonia case 6 show a probable macrophage (arrow) at the luminal epithelial surface. (L and M) MV and CD150 positivity is seen at the luminal aspect of the epithelium (L and M); in the CD150 preparation, the epithelium is artifactually detached from its basement membrane through microtome sectioning (M). (N and O) Similar patterns of MV (N) and CD68 (O) positivity at the alveolar luminal surface in late measles pneumonia case 20. (P) In a further late case of measles pneumonia (case 17), MV^+^ staining is seen in an MGC. (Q to S) This is also observed with CD68 (Q), Iba1 (R), and CD150 (S) staining. Original magnifications: panels A and B, ×500; panel D, ×640; panel E, ×250; panel F, ×400; panels G to J, ×100; panel K, ×1,000; panel L, ×600; panel panels M to O, ×400; panels P, Q, R, and S, ×40.

In contrast to the paucity of bronchial and bronchiolar infection in the prodromal phase, all patients with pneumonia in the established phase had marked MV infection in alveoli (Fig. 5). Normal architecture of alveoli was observed in a prodromal case 1 (Fig. 5A), whereas in other cases (case 10, Fig. 5B to F; case 13, Fig. 5G to J), extensive pathology was observed. Alveoli contained many infiltrating inflammatory cells (Fig. 5B), and MV antigen either partially (Fig. 5C) or totally (Fig. 5H) outlined alveolar walls. Positive staining for both CD68 (Fig. 5D) and CD11c (Fig. 5E) was present in locations basal to and within alveolar epithelium. CD150^+^ cells were present in the interstitium and formed part of the alveolar lining (Fig. 5F). In cases 10, 13, and 20, hyaline membranes were found to some degree; where the pneumonia was severe, the level of hyaline membrane formation was proportionate (case 13; Fig. 5G). Hyaline membranes showed focal MV antigen positivity (case 13; Fig. 5H), were pan-cytokeratin positive (Fig. 5I), and were infiltrated by CD11c-expressing cells (Fig. 5J). MV antigen containing MGCs lined alveoli, and serial sections expressed both CD68^+^ cells (Fig. 5C and D) and, to an even greater extent, CD11c^+^ cells (Fig. 5E). Infected mononuclear cells had the phenotype of DCs or macrophages and stained variably for CD150. In one case of established disease (case 6), there was a superficial macrophage stained by H&E (Fig. 5K), MV antigen was present across the luminal surface of the bronchiolar epithelium (Fig. 5L). In the same case, CD150 staining was present on superficial bronchiolar epithelium toward the lumen (Fig. 5M).

### (iv) Late (cases 17 to 23).

Coexisting immune system diseases (lymphoma, leukemia, or genetic immunodeficiency) were present in all seven late cases (Table 1), and disseminated measles involved several organs. The majority of intraepithelial MV-infected mononuclear cells in the lung were macrophages or DCs, mirroring the established cases. In a late case (case 20), MV-positive cells are seen against a carnified background, most probably coating an alveolus (Fig. 5N); the area is shown with respect to CD68 staining in a serial section (Fig. 5O). In a different late case (case 17), an MGC is present in serial sections as MV^+^ (Fig. 5P), CD68^+^ (Fig. 5Q), and Iba1^+^ (Fig. 5R). Taken together, these data confirm that, irrespective of the phase of disease, MV infection of an epithelium predominantly took place in immune system cells and that limited infection of epithelial cells could be demonstrated.

### MV predominantly infects cells of the immune system, irrespective of the organ involved.

**FIG 6:**
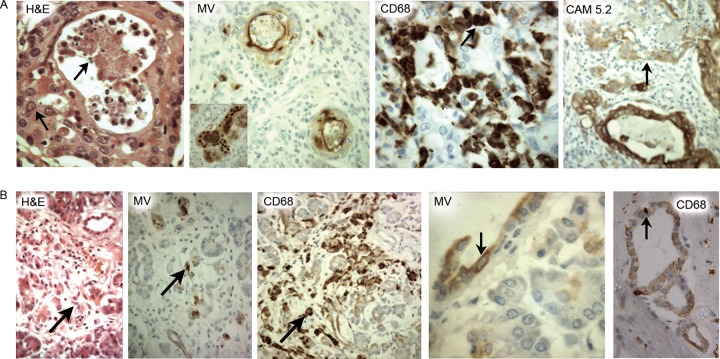
Immunohistochemical analysis of host responses in salivary gland epithelia in an early-measles case and a late-measles case. The images show an intercalated duct in the salivary gland in late case 17 (A) and early case 5 (B) with macrophages (inset) and MV positivity at the epithelial luminal surface. CD68 staining shows positivity at the epithelial luminal surface and in the body of the salivary gland. Note the low level of CAM 5.2 expression in the infected duct (arrow in the rightmost panel in Fig. 6A). Original magnifications: panel A, ×1,000 (H&E stain), ×500 (MV; inset, ×640), ×640 (CD68), and ×310 (CAM 5.2); panel B, ×400 (H&E; MV and CD68), ×250 (CD68), and ×100 MV (fourth image from the left).

MV antigen was demonstrated in the lung in 18 of the cases (Table 2). However, in six of those patients who had coexisting disease of the immune system or congenital heart disease, the infection was disseminated, variously involving salivary gland, pancreas, liver, and adrenal tissue (Table 2). In the salivary gland of late case 17, MV antigen was present in the lining of intercalated ducts where the epithelium was fragmented or lost, and the virus was closely associated with CD68^+^ macrophages (Fig. 6A). In the pancreas of early-measles case 5, viral antigen was observed in intercalated duct epithelium and in surrounding connective tissue but not in acini or islets (Fig. 6B). CD68^+^ cells were also seen in the same locations in those tissues. In the adrenal gland, infection was restricted to the cortex, with MV-infected foci showing hemorrhagic necrosis and infiltration by CD68-expressing cells (data not shown).

**FIG 7:**
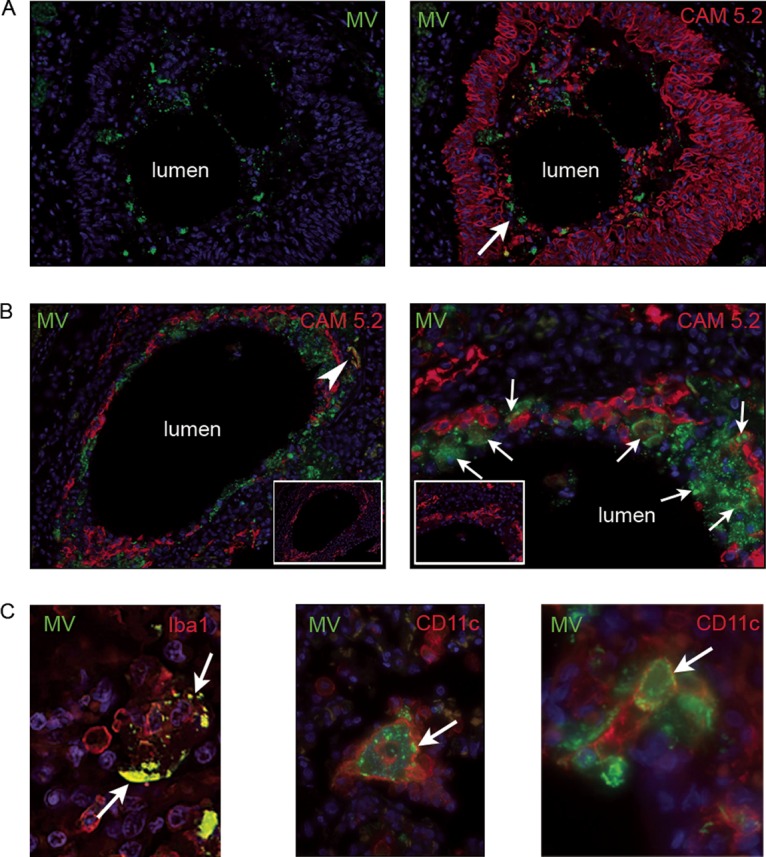
Identification of MV-infected cell types in the lung of established-measles cases and a late case of measles pneumonia. (A) Fluorescent microscopic images from a bronchiole (case 7) labeled with MV (green) and CAM 5.2 (red) antibodies and nuclei labeled with DAPI (blue). MV positivity is seen within the lumen, distinct from the luminal epithelial surface. CAM 5.2^+^ cytokeratin fragments are also present in the lumen, a few of which colocalized with MV (arrow). (B) Fluorescent microscopic images (case 10) of a bronchus labeled with MV (green) and CAM 5.2 (red) antibodies with nuclei visualized using DAPI (blue) (inset, CAM 5.2 [red]). The disrupted epithelium is shown by the focally discontinuous CAM 5.2 positivity around the bronchial margin. MV positivity is present predominantly at the luminal aspect of the fragmented epithelium. An MV nucleoprotein is seen to colocalize with a number of CAM 5.2^+^ epithelial cells (arrows) in the right image. (C) CLSM images from lung sections labeled with MV (green), Iba1 (red), and CD11c (red) antibodies and DAPI-labeled nuclei (blue). MV colocalized with Iba1 (arrow) in an MGC in established-measles pneumonia (left image, case 10). MV colocalized with CD11c (arrow) in established (middle image, case 13) and late (right image, case 20) measles pneumonia. Original magnifications: panel A, ×200; panel B, ×200 (left image) and ×400 (right image); panel C, ×400 (MV and Iba1) and ×630 (CD11c).

Due to the importance of our observations for the understanding of measles pathogenesis, transmission, and viral receptor usage, sections from nine established and late cases were dually labeled with antibodies specific for MV and epithelial cells (CAM 5.2 and AE1/AE3, representing a mixture of two different clones of anti-cytokeratin monoclonal antibodies with a broad spectrum of reactivity) or a panel of immune cell proteins, including CD150, CD11c, CD68, Iba1, CD3, and CD20. Confocal laser scanning microscopy (CLSM) and UV microscopy were used to visualize the double-stained sections (Fig. 7) in lung sections from the established-measles cases (cases 7, 10, and 13) and the late-measles cases (case 20). Immunoperoxidase staining confirmed results obtained by dual labeling. In established-measles and late-measles cases involving pneumonia, foci of infection in bronchi and bronchioles were rare but, when present, were found within the lumen, close to the luminal epithelial margin (Fig. 7A). Cytokeratin fragments, presumably derived from sloughed epithelial cells, were also seen in the lumen (Fig. 7A, arrow), often in areas also staining positively for MV. In infected alveoli of established-measles cases, the epithelium was largely necrotic and fragments of intact detached epithelium were seen together with fragments of cytokeratin (Fig. 7B). Low numbers of MV-infected epithelial cells were present within the epithelium (Fig. 7B, arrows), and MV antigen was also observed to colocalize with cytokeratin fragments. However, in these same areas, the predominant colocalization of MV was with CD11c^+^ and Iba1^+^ cells (Fig. 7C). These cells were multinucleated or had the morphology of macrophages or DCs. MGCs were, in general, negative for cytokeratin, although small fragments of cytokeratin were occasionally present within their cytoplasm (data not shown).

### MV in a Schwarz vaccine-induced case was closely associated with DCs and macrophages.

A case of vaccine-induced measles (case 18) was particularly significant and important because, in theory, the virus could use CD46, CD150, and nectin-4 as cellular receptors. The patient had a genetic disorder reported as dysgammaglobulinemia (23) and died with giant cell pneumonia and disseminated measles 7 weeks after measles vaccination. The diagnosis of measles was made by the pathologist, and MV was isolated from tissue at the time of necropsy and further characterized as derived from the Schwarz vaccine virus. This MV strain (Hu2) was subsequently used in a comparative study of sequence divergence of MV hemagglutinin during natural evolution and adaptation to cell culture (24). Nucleotide sequencing showed that this strain retained the amino acid residues crucial for interaction with CD46 (25) and CD150 (26, 27). Pathological analysis of tissues from this case and staining for MV showed that MV antigen was present in many organs, including lung, liver, pancreas, adrenal, bone marrow, lymph nodes (LNs), and thymus. In the respiratory tract, despite the parenteral route of immunization, both lungs were heavily infected.

**FIG 8:**
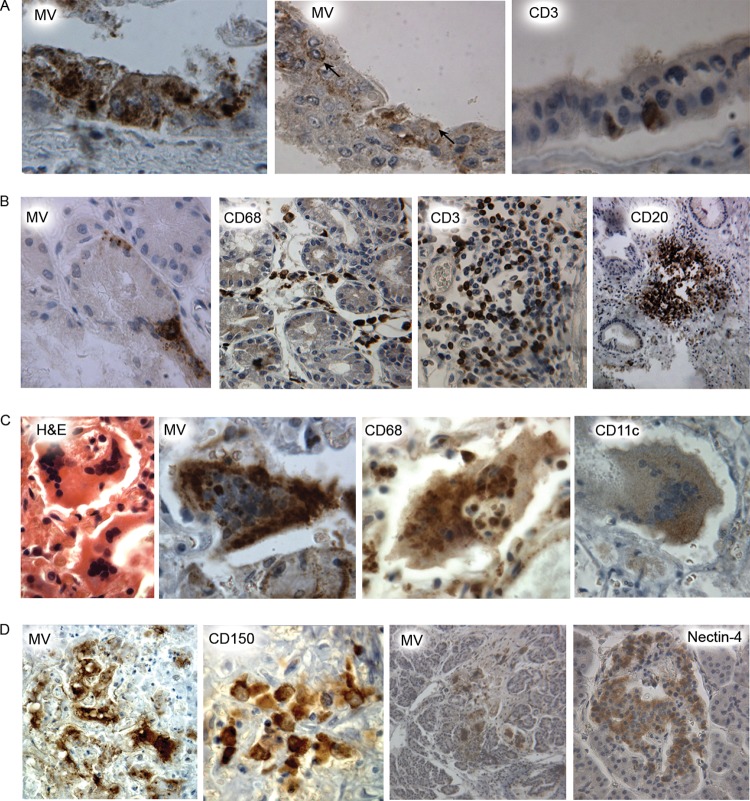
Immunohistochemical analysis of trachea, liver, lung, and pancreas tissue from a Schwarz vaccine-induced case of measles (case 18). (A) The epithelial layer in the trachea. The three images show MV antigen (left and middle) and CD3 (right) staining in the epithelial layer of the lower trachea (arrows) and demonstrate a rare epithelial infection. (B) Subepithelial infection in the trachea. The four images show subepithelial tissues of the lower trachea stained for MV, CD68, CD3, and CD20 with substantial MV infection and substantial infiltration of immune cells. (C) MGCs in the lung consisted of MV-infected CD68^+^-expressing cells and CD11c-expressing cells. In the H&E and CD68 preparations, the MGCs contain cells whose nuclei do not form part of the central nuclear cluster. (D) The first and second images at the left represent liver tissue showing MV antigen-positive cells and CD150-expressing cells, respectively. The right two images are serial sections of pancreas tissue, with MV-infected cells and nectin-4^+^ cells showing different localization patterns. Original magnifications: panel A, ×400 (MV, MV, and CD3); panel B, ×400 (MV), ×250 (CD68 and CD3), and ×100 (CD20); panel C, ×800 (H&E), ×640 (MV and CD11c), and ×400 (CD68); panel D, ×400 (MV), ×800 (CD150), and ×100 (MV and nectin-4).

The trachea was chronically inflamed and rare, but clear infection of the epithelium was observed (Fig. 8A, left hand panel). Sections from the trachea were stained for MV, CD20, CD3, and CD68 antigens (Fig. 8A and B). CD20-expressing cells were infected in the subepithelium, whilst CD3-expressing cells were infected in the basal epithelium and in the subepithelium. CD68-expressing cells were infected and occasionally positive in the epithelium but were more predominant in the subepithelium and in the mucus glands. Results of staining for CD11c, CD150, nectin-4, and CD46 were negative. MV infection predominantly involved subepithelial cells that express markers of the immune system (Fig. 8B). MV antigen MGCs were present in alveoli and interstitia of the lung (Fig. 8C). MGCs stained positively for MV, CD11c and/or CD68, and Iba1 and variably for CD150.

The inflammatory response within the lung was predominantly mononuclear, with CD68^+^ and CD11c^+^ cells in alveoli and interstitia (Fig. 8C). In noninfected liver tissue, cells which stained positively for CD11c, CD68, and CD150 lined sinusoids and had the morphology of Kuppfer cells. Surrounding MV-infected foci, a narrow zone with negative CAM 5.2 staining was observed, indicating some epithelial cell loss (data not shown). MV antigen was observed in portal tracts with an associated infiltration of CD11c-expressing and CD150-expressing cells (Fig. 8D, left two panels). The pancreas showed MV antigen and nectin-4-expressing cells (Fig. 8D, panels at the right). Nectin-4 staining was positive in the islets. MV antigen cells were never positive in the islets but were predominantly present in the connective tissue between the pancreatic acini. MV infection was not observed in epithelial cells in either the liver or the pancreas. These data suggest that even in vaccine-induced measles, in which the virus can be demonstrated to be capable of using CD46, CD150, and nectin-4 as cellular receptors *in vitro*; the *in vivo* situation in the natural host differs markedly, with the cell types expressing CD150 being those predominantly infected (28, 29).

## DISCUSSION

The data from this unique collection of tissues from measles cases indicate that MV largely infects cells of the immune system independently of organ or stage of the disease. Infection of macrophages and DCs, within epithelia or adherent to the epithelial surface, was the most striking finding. Of particular relevance to one hypothesis pertaining to measles transmission was the observation that very few MV-infected cells were present in the tracheal and bronchial and bronchiolar epithelium (30). Pronounced alveolar infection was observed in all fatal early, established, and late cases, including the parenterally induced vaccine case, suggesting that virus-infected cells were present in these tissues throughout the disease process. Cells of an immune system lineage were frequently observed in MV-positive foci in bronchi, bronchioles, and alveoli, and dual labeling with myeloid cell markers indicated that MV protein(s) colocalized with these cells. These observations show that in this subset of mostly fatal human cases, MV infection of the respiratory epithelium was largely restricted to cells of the immune system. Although a low-level infection of respiratory epithelial cells was observed, it is possible that the true level of epithelial cell infection in measles was higher, as we were unable to obtain tissues from the nasal conchae, hard and soft palate, and nasal septum, which have been shown to be heavily infected in the macaque (31, 32).

To date, no biological variations in host pathological responses have been shown to be associated with different MV genotypes, although depletion of MV glycoprotein-specific antibodies from human sera with specific MV strains has revealed genotype-specific neutralizing antibodies in early convalescent-phase serum samples (33). In the present study, despite the span of 38 years in the dates of presentation, the geographical spread, and the variety of coexisting diseases, the cellular pathological responses were identical in all cases. Molecular epidemiological studies have shown that wild-type MV genotypes differ over time and across continents, but in outbreaks of measles, a single serotype has been identified in each epidemic (34). Two of the patients (cases 5 and 17) died as a result of the outbreak in São Paulo, Brazil, in 1997 from which the MV genotype D6 was isolated (35), and the samples from the two South Korean patients (cases 2 and 3) were from the measles epidemic in 2000 from which the H1 genotype was isolated (36). The nine Ivory Coast patients (cases 7 to 16) were most likely infected by strains corresponding to the African B clade of MV genotypes as this has been shown to circulate in the Côte d’Ivoire (37). Thus, in this case series, there is constancy of cellular pathological response despite evidence of viral genotype variation.

In the one vaccine-induced case (case 18) in which the virus, according to its nucleotide sequence, was capable of using CD46, CD150, and nectin-4 as cellular receptors, infected cells were predominantly of the immune lineage, suggesting that even laboratory-adapted strains of MV preferentially use CD150 as a receptor *in vivo*. However, in contrast to the other human cases associated with wild-type MVs, isolated infected columnar epithelial cells were observed in the trachea of the vaccine case together with infected CD3-expressing T cells. However, the observations in this human case mirror those in macaques (28, 29). Similar cases of vaccine-induced fatal disease in children with an immune disorder have been published previously (38, 39), but to our knowledge there have been no reports of MV receptor status in infected cells in such cases.

This collection of measles cases can be divided into two categories with respect to coexisting diseases (Table 1): first, those cases in which measles was coincidental and not a major factor in death; second, those cases in which measles was the immediate cause of death. In the coincidental category, e.g., patients with congenital heart disease, measles infection, while possibly contributing to death, was not the most important factor. For example, in one prodromal case (case 1), death followed cardiac surgery and the diagnosis of measles was made by the pathologist. These cases represent rare opportunities to observe MV-host interactions in tissues from patients in whom measles was coincidental and therefore presumably mirror the usual nonfatal pattern of this infection. In the second category, patients had genetic, neoplastic, or infective disease of the immune system. Measles was the major cause of death in these patients, and in some the clinical course was prolonged by the involvement of unusual sites of infection. In this second category, the host cellular response, albeit more intense, was, surprisingly, the same with respect to the identity and distribution of the inflammatory cells as that seen in cases in the first category, in which measles was coincidental to death. In neither category can the viral load be estimated. However, more MV-infected cells were observed in those patients who had preexisting immune disease. In the latter group, measles was stated as the cause of death, but the exact mechanism by which the virus induced a lethal outcome remains uncertain. The alveolar epithelium was often disrupted, and cytokeratin fragments were present in the interstitium. MV antigen was observed at the luminal surface of the alveolar epithelium in these cases. Irrespective of the deleterious effect of the virus on the immune system and the underlying diseases, the widespread loss of intercellular cohesion between adjacent bronchiolar and alveolar epithelial cells associated with the measles infection must have been at least a factor that contributed to death. The physiological state in which adjacent respiratory epithelial cells are tethered is E-cadherin dependent, and disruption of this bond leads to barrier malfunction (40). However, E-cadherin is responsible not only for epithelial cell binding but also for the bond between DCs and epithelial cells (41). MV infection of DCs produces lowered levels of E-cadherin, and it has been suggested that this loss of E-cadherin may have relevance for DC migration (42). It may also be relevant to respiratory epithelial cell loss of cohesion.

Nonhuman primates can be infected with a recombinant (r) wild-type MV, which does not bind the nectin-4 receptor, and they fail to shed infectious virus (43, 44). Based on these macaque models, this receptor is therefore proposed to play a significant role in amplification and transmission of the virus between patients but not during the early stages of infection. Nectin-4 is a cellular adhesion molecule closely related to the three other known nectins (PVRL-1, PVRL-2, and PVRL-3) and is localized to adherens junctions at the basolateral side of cell boundaries. This molecule is highly expressed in embryo- and placenta-derived tissues, but the normal distribution of nectin-4 in adult tissues is limited to the trachea, the appendix, and the placenta, and its expression in the lung is very low and has been shown to be a tumor marker (45). In our study, pancreatic cells which strongly expressed nectin-4 were not infected. Low-level expression was reported by Noyce et al. (15) in tonsillar epithelium and on reactive pneumocytes in the lung. We were able to show only weak staining for nectin-4 in the tracheal epithelium which did not colocalize with MV infection. In monkey models, de Swart and coworkers rarely observed epithelial damage in the tracheal epithelium in contrast to the very common disruption of epithelia in the lung and nasal cavity. In animals sampled at late time points after infection, infection of tracheal epithelium was usually absent, i.e., had likely been cleared by the virus-specific immune response (R. L. de Swart, personal communication). This may perhaps explain why infection of these cells is not detectable in human tissues. Clearly, the exact role of nectin-4 in measles pathogenesis in the human remains to be elucidated, especially with respect to how the extensive infiltration of MV-infected immune cells and concomitant epithelium disruption impinges on nectin-4 expression levels on adjacent epithelial cells.

Classical reports on MV pathogenesis showed the involvement of tonsil, lymphoid tissue of the appendix, LNs, skin, and pharyngeal mucosa in the pathological process and established the presence of MGCs in lung and lymphoid tissues. These reports emphasized the prominence of mononuclear cells in the inflammatory response (46, 47). However, in general, although the presence of MV proteins and/or genome in tissues was demonstrated in these studies, only classical staining techniques were used to assess the identity of infected host cells. Those few studies of MV pathogenesis in which human-cell-specific markers and protein markers were used have been restricted to lymphocyte and cytokine identification (48–52).

While our data support the idea that the infection of immune cells is the most important factor in early-measles pathogenesis, a finding also demonstrated in macaque models, they also appear to be the main cell type that is infected in the later stages of disease in these human cases. In other viral infections, DCs have been shown to process viral antigen from apoptotic epithelial cells (53). Canine distemper virus (CDV) and rinderpest virus, which are closely related to MV, also target the immune system (54–56). However, in cases of distemper in the ferret model and dogs (57), epithelial cell infection is consistently described, with some studies reporting that this occurs at a later stage than infection of circulating lymphocytes and lymphoid tissues (57). Furthermore, a natural outbreak of CDV in monkeys showed predominance of immune system cell infection but also occasional infection of nectin-4-positive cells in the bronchi and bronchioles (58).

Respiratory epithelial cell infection has been described previously in early studies performed with MV in a rhesus monkey model (59), but dual-labeling experiments with epithelial cell markers were not performed. In a macaque model of measles, the main MV cellular targets were DCs and CD150^+^ lymphocytes and dual labeling using a cytokeratin marker proved that small numbers of epithelial cells were infected in the respiratory tract. A recent paper describing a study that used the macaque model (30) demonstrated a difference in pathologies between wild-type MV and nectin-4 “blind” virus, with only the wild-type virus being able to infect columnar epithelial cells in the trachea. In contrast to the human cases studied here, a strength of the macaque model is the known interval between infection and tissue sampling. Minor differences in the observed pathologies in the human and macaque cases may be real or may be due to methodological differences such as the use of viruses with fluorescent reporters, which results in the exquisite sensitivity.

In conclusion, our data obtained from a limited set of human measles cases with confounding factors indicate that the majority of cells infected in epithelia in a wide range of organs were cells of the immune system. The infected cells were predominantly of a myeloid lineage. Given the possible role of MV-infected DCs in the immunosuppression associated with measles (60), the data relating to infected DCs within the respiratory epithelium in this study may be significant. A role has been proposed for nectin-4-mediated epithelial cell infection in the amplification of virus for transmission. However, the infection in these 23 human cases is associated with extensive noninfective epithelial cell necrosis, with only small numbers of infected epithelial cells observed. These observations augment our understanding of measles pathogenesis in humans and highlight the necessity for further investigations into the *in vivo* expression of nectin-4 in human tissues and its role during natural measles.

## MATERIALS AND METHODS

### Patients.

Blocks of formalin-fixed tissue, already processed using paraffin, were obtained from archival cases of measles from Africa, Asia, Europe, and North and South America (see Table 1 for clinical details and categorization of the phase of measles). Ethics approval for research had already been obtained in each country of origin, and the study was approved by the Northern Ireland NHS Research Ethics Committee (reference no. 4/03) and the Royal Victoria Hospital Research Governance Committee (reference number RHGH 1000127).

### Tissue sectioning and staining.

Sections were cut at 4 µm on a rotary microtone (Reichert-Jung), mounted on glass slides, and stained with H&E. After microscopic screening, sections that were histologically abnormal were stained for MV proteins.

### Immunohistochemistry.

Immunohistochemistry analyses were performed on formalin-fixed, paraffin-embedded sections that were mounted on activated glass slides using the following antibodies and dilutions: monoclonal antibody to measles (clone 49–21; Immunologicals Direct) (1:2,500), polyclonal antibody to measles nucleoprotein (Novus Biologicals, Inc.) (1:4,000), monoclonal antibody to macrophages (CD68, clone PGM1; Dako) (1:100) or polyclonal antibody to Iba1 (Wako, Japan) (1:100), histiocytes and dendritic cells (CD11c, clone 5D11; Novocastra) (1:50), T cells (CD3, clone F7.2.38; Dako) (1:50), B cells (CD20, clone L26; Dako) (1:50), and monoclonal antibodies to cytokeratins (clone CAM 5.2 [Becton, Dickinson] [neat] and clone AE1/AE3 [Dako] [1:100]) or polyclonal antibody to cytokeratins (polyCK; Novocastra) (1:100). Antibodies to the MV receptors were polyclonal anti-CD46 (gift from F. Wild, Institut Pasteur de Lyon, France) (1:200) and monoclonal antibody to SLAM-F1 (CD150; Novocastra) (1:50). Anti-PVRL-4 (nectin-4) was a rabbit antiserum (HPA010775; Sigma) diluted 1:400.

After sections had been dewaxed and rehydrated, endogenous peroxidase was blocked by 10 min of incubation in 3% (vol/vol) hydrogen peroxide in methanol at room temperature followed by a 5-min wash in running tap water. Sections were then subjected to antigen retrieval by microwaving for 20 min at 700 W in 0.01 M sodium citrate (pH 6.0) or by pressure-cooking at full steam for 3 min in 0.01 M Tris-EDTA buffer (pH 9.0). After a further wash in running tap water for 5 min, sections were incubated in primary antibodies for 16 h at 4°C. After further Tris-buffered saline (TBS) washes, bound antibodies were detected by the use of anti-mouse EnVision peroxidase (monoclonal antibodies) or anti-rabbit EnVision peroxidase (polyclonal antibodies). Peroxidase was visualized with DAB (diaminobenzidine) (DakoCytomation; EnVision system-horseradish peroxidase; catalog no. K4007). Sections were then counterstained in hematoxylin, dehydrated, and mounted. Negative and positive controls were done with each batch of antibody.

### Dual indirect immunofluorescence.

In selected tissue blocks, dual immunofluorescence labeling was used to map the cell distribution of virus. Following antigen retrieval, sections were incubated overnight at 4°C in a mix of MV antibodies and one of the cell-marking monoclonal antibodies. Following washes in TBS, antibody binding sites were detected by incubation in a mix of goat anti-rabbit Alexa 568 and goat anti-mouse Alexa 488 (Invitrogen, Dublin, Ireland) (both 1:500) for 2 h at 37°C. After final rinses in TBS, sections were mounted in 4′,6-diamidino-2-phenylindole (DAPI) Vectashield (Vector Laboratories, Dublin, Ireland). The following dual-labeling experiments were done: MV protein(s) with CD68, Iba1, CD11c, CD3, CD150, CAM 5.2, or AE1/AE3.

### Microscopy.

A Leica bright-field microscope (Leica Aristoplan, with digital camera using Leica 1000 software) was used to assess and acquire images from H&E-stained and peroxidase-stained sections. Immunofluorescent stains were analyzed and images acquired and processed using a Leica TCS SP2 system, a Leica TSC SP5 system, and UV CLSM acquisition software and a Leica DM600B UV microscope with FW4000 acquisition software. In the CLSM experiments, the red and green channels were imaged sequentially.

### Evaluation of immunohistochemistry sections.

Sections were scored by two persons according to density of cellular staining, numbers of cells, and area positively stained within a section. In the case of cytokeratin immunohistochemistry, loss of cytokeratin in a site that should have been cytokeratin positive was recorded.
